# Triazoles Versus Echinocandins for the First-Line Treatment of Invasive Pulmonary Aspergillosis: A Propensity Score–Weighted Multicenter Study

**DOI:** 10.1093/ofid/ofaf709

**Published:** 2025-11-18

**Authors:** Stefan Hatzl, Christina Geiger, Lisa Kriegl, Andreas Reinisch, Markus Keldorfer, Albert Wölfler, Markus Wallner, Dirk von Lewinski, Philipp Eller, Robert Krause

**Affiliations:** Department of Internal Medicine, Intensive Care Unit, Medical University of Graz, Graz, Austria; BioTechMed-Graz, Graz, Austria; Department of Internal Medicine, Division of Infectious Diseases, Medical University of Graz, Graz, Austria; BioTechMed-Graz, Graz, Austria; Department of Internal Medicine, Division of Infectious Diseases, Medical University of Graz, Graz, Austria; Department of Internal Medicine, Division of Hematology, Medical University of Graz, Graz, Austria; Department of Blood Group Serology and Transfusion Medicine, Medical University of Graz, Graz, Austria; Department of Pediatrics and Adolescent Medicine, Pediatric Intensive Care Unit, Medical University of Graz, Graz, Austria; Department of Internal Medicine, Division of Hematology, Medical University of Graz, Graz, Austria; Department of Internal Medicine, Division of Cardiology, Medical University of Graz, Graz, Austria; Department of Internal Medicine, Division of Cardiology, Medical University of Graz, Graz, Austria; Department of Internal Medicine, Intensive Care Unit, Medical University of Graz, Graz, Austria; BioTechMed-Graz, Graz, Austria; Department of Internal Medicine, Division of Infectious Diseases, Medical University of Graz, Graz, Austria

**Keywords:** aspergillosis, critically ill, echinocandins, ICU, immunocompromised, triazoles

## Abstract

**Background:**

Triazoles (eg, isavuconazole, posaconazole, voriconazole) are the first-line treatment for invasive pulmonary aspergillosis (IPA), but their use can be limited by drug interactions, hepatotoxicity, and emerging azole resistance. Recent guidelines recommend echinocandins as a safe and well-tolerated first-line alternative.

**Methods:**

In this multicenter observational study across 9 treatment centers, we included all consecutive patients with IPA from 1 January 2014 to 1 June 2024. We compared 30-day overall survival (OS) as the primary outcome and 90-day OS as the secondary outcome between patients receiving triazoles versus echinocandins as first-line therapy. Propensity score adjustment was applied to address baseline characteristic imbalances.

**Results:**

We included 177 patients, of whom 153 (86%) received triazoles as first-line therapy (69 voriconazole, 54 isavuconazole, 30 posaconazole). Before propensity score adjustment, patients treated with echinocandins were significantly sicker, with higher rates of intensive care treatment (95% vs 75%, *P* = .002). In both crude and propensity score–adjusted analyses, 30-day mortality was significantly higher in the echinocandin group compared to the triazole group (63% [95% CI 55–70] vs 30% [95% CI 13–50], *P* < .001). Even switching from echinocandins after initial treatment failure to salvage therapy did not mitigate the negative impact on OS. Switching from triazoles (28/153 cases) was mainly due to tolerability concerns, while echinocandins (12 cases) were switched for treatment failure.

**Conclusions:**

Using echinocandins as alternative first-line treatment for IPA may result in poorer survival outcomes and should be approached with caution until further trials confirm our findings.

## Teaser

In this multicenter study including 177 patients with invasive pulmonary aspergillosis, first-line echinocandin use was associated with higher 30-day mortality than triazoles. These findings suggest caution with first-line use of echinocandins.

Invasive pulmonary aspergillosis (IPA) is a life-threatening fungal infection of the lungs caused by *Aspergillus* species, primarily affecting immunocompromised and critically ill patients [1, 2]. Current treatment options for IPA are limited to 3 main classes of antifungal drugs. Triazoles (eg, voriconazole, posaconazole, isavuconazole) and polyenes (mainly liposomal amphotericin B) are recommended as primary therapies with differences in grading, while echinocandins (caspofungin, anidulafungin, micafungin) are considered as alternatives in eg, cases with azole resistant *Aspergillus* species, though rarely as monotherapy [3–5]. Although newer antifungal agents such as olorfim and fosmanogepix have shown potential, they are not yet widely implemented in clinical practice [6].

Recent data indicate that triazoles are the most prescribed initial treatment for IPA (67% of cases), while echinocandins as monotherapy were used in only 5% of cases [7]. The choice of antifungal treatment requires careful evaluation of drug efficacy, pharmacokinetics, tissue penetration, side effects including concerns of triazole-related liver toxicity in critically ill patients, and incidence of antifungal resistance. Echinocandins are gaining increased attention due to the growing resistance to azoles in *Aspergillus fumigatus* and the safety concerns associated with triazoles, although newer azoles are better tolerated [8–10].

However, there is a lack of randomized clinical trials comparing echinocandins directly to other antifungal agents for IPA treatment. Previous studies report success rates of echinocandins in IPA treatment ranging from 30% to 55%, primarily in patients with hematological malignancies. Recent IPA treatment guidelines still recommend echinocandins as an alternative to triazoles [11–14].

To assess the role of echinocandins in IPA, our multicenter cohort study aimed to compare the efficacy of triazoles versus echinocandins in treatment of IPA using propensity score–weighted analyses.

## METHODS

### Data Source

For data extraction, we used openMedocs (SAP), the electronic health record system of the Steiermärkische Krankenanstalten Gesellschaft (KAGes), which provides patient-level data for all hospitals within the network. Data were uniformly collected following established protocols, and laboratory, clinical, and radiological information were retrieved from openMedocs and entered into a predefined electronic case report form (eCRF) using REDCap [15, 16].

### Patient Cohort

We conducted a retrospective multicenter cohort study across 9 treatment centers, including all consecutive adult patients (18 years or older) diagnosed with IPA. (Supplementary Figure 1) The study period extended from 1 January 2014 to 1 June 2024 [15, 16]. The local ethics committee (EK:32-302ex19/20) approved the study, which was conducted in accordance with the Declaration of Helsinki principles. Patient comorbidities were assessed using international classification of diseases (ICD-10) codes if documented in any medical report within 12 months prior to study inclusion. For the final analysis, we included only patients with IPA either treated with an echinocandin or a triazole based on decisions of the treating physicians.

Invasive pulmonary aspergillosis was defined based on the updated European Organization for Research and Treatment of Cancer/Mycosis Study Group (EORTC-MSG) criteria or, for patients admitted to intensive care units (ICUs) who lacked EORTC host factors, the FUNDICU (Invasive Fungal Diseases in Adult Patients in Intensive Care Units) criteria. Patients who did not meet these criteria but received a diagnosis of IPA from an infectious diseases specialist were classified as having clinical IPA [1, 2, 17].

### Antifungal Treatment Exposure

We defined antifungal treatment started on the IPA diagnosis date as first-line therapy, classifying patients by agent: triazoles (voriconazole, isavuconazole, posaconazole) or echinocandins (caspofungin, anidulafungin). Exposure was guideline-concordant if a triazole was used first-line and alternative if an echinocandin was used without guideline indication. Treatment duration, administration route, and any switches to second-line or salvage therapy were recorded. Switches were considered treatment failure, defined by lack of IPA resolution on imaging, failure of clinical improvement, or adverse effects, including uncontrollable drug trough levels.

### Statistical Analysis

All statistical analyses were performed using Stata (Windows version 18.1) and R version 4.0.5. Baseline differences between the triazole and echinocandin groups were assessed using rank-sum, χ^2^, and Fisher's exact tests as appropriate. Standardized mean differences (SMDs) quantified the magnitude of differences, with SMD ≥ 0.30 considered significant.

To adjust for baseline differences, propensity score analyses were conducted using a multivariable logistic regression model with antifungal treatment as the outcome. The model included up to 10 predictor variables, ensuring at least 3 events per variable. Variables with *P* ≤ .15 or SMD ≥ 0.30 were included. Propensity scores were transformed into inverse probability of treatment weights (IPTW) using the average treatment effect formula (triazole vs echinocandin). Balance diagnostics were reassessed with SMDs and *P* values after IPTW adjustment [18, 19].

No patients were excluded due to incomplete data. All baseline variables were fully observed except for procalcitonin and bronchoalveolar lavage galactomannan (BAL GM), for which missing values were imputed using chained equations. After IPTW adjustment, between-group comparisons were revisited. Sensitivity analyses included trimming IPTW by excluding patients with weights outside the 1st and 99th percentiles.

The primary outcome, 30-day overall survival (OS) after IPA treatment initiation, was estimated using Kaplan–Meier survival curves and compared with the log-rank test. Independent predictors of 30-day OS were identified through univariable and multivariable Cox proportional hazards regression. Survival time was inflated by one day for patients who died on the day of IPA diagnosis (*n* = 4).

All analyses considered *P* < .05 statistically significant. The full dataset and analysis codes are available upon request.

### Sensitivity Analysis

For sensitivity analysis, we applied a trimmed IPTW approach to address potential instability from extreme weights. Observations with propensity scores in the tails of the distribution were excluded, trimming those below the 5th percentile and above the 95th percentile to minimize the influence of outliers. This trimmed IPTW was recalculated and applied to re-weight the dataset, allowing us to assess the robustness of our findings under a reduced influence of extreme propensity scores.

## RESULTS

### Study Cohort

Over the study period, 177 patients were analyzed, with 153 in the triazole group and 24 in the echinocandin group (Supplementary Figure 2). In the triazole group, 69 received voriconazole, 54 received isavuconazole, and 30 patients received posaconazole. In the echinocandin group, 16 patients received anidulafungin and 8 received caspofungin. The median age was 63 years [55–71] across all patients, with no significant difference between groups. A total of 51/177 (29%) patients were female, with a higher proportion in the triazole group (31%). Laboratory findings were generally similar between groups, except for higher procalcitonin (PCT) levels in the echinocandin group.

Median serum GM was 0.51 ODI [0.14–0.90], while BAL GM was notably higher in the echinocandin group (7.38) compared to the triazole group (4.29) (*P* = .014). The echinocandin group also had significantly higher serum β-D-glucan levels (median 183.6 pg/mL) versus the triazole group (75.2 pg/mL) (*P* = .044). Asp-PCR in BALF was positive in 70% of patients, with no group difference.

Among all patients, 77% required ICU admission, with a significantly higher proportion in the echinocandin group (95%) compared to the triazole group (75%) (*P* = .020). The median APACHE II score was 21 [7–30], with the echinocandin group scoring higher at 30 [23–33] compared to 19 [3–29] in the triazole group (*P* = .002). Sequential organ failure assessment (SOFA) scores were also higher in the echinocandin group (median of 10 [7–12]) versus the triazole group (7 [4–8]) (*P* < .001). The median paO2/FiO2 ratio was 126 [81–268] across the cohort, showing no significant differences between groups (*P* = .165). (Table 1)

**Table 1.** Baseline Characteristics

Variable	Missing, *n* (%)	Overall (*n* = 177)	Triazole (*n* = 153)	Echinocandin (*n* = 24)	*P*	*P*_IPTW_
Age (years)	0 (0%)	63 [55–71]	63 [54–71]	64 [59–71]	.649	.091
Female	0 (0%)	51 (29%)	3 (13%)	48 (31%)	.058	.495
BMI (kg/m^2^)	0 (0%)	25.4 [22.7–28.7]	25.4 [22.6–28.7]	26.0 [24.2–27.6]	.538	.984
Laboratory findings						
Leukocytes [G/L]	0 (0%)	9.4 [5.2–13.8]	9.4 [5.0–13.6]	9.7 [6.4 -15.1]	.494	.338
Neutrophils [G/L]	0 (0%)	8.0 [4.0–11.9]	7.8 [3.5–11.8]	8.8 [4.7–13.3]	.394	.342
Lymphocytes [G/L]	0 (0%)	0.6 [0.3–1.0]	0.6 [0.3–1.0]	0.6 [0.3–1.2]	.758	.278
Hemoglobin [g/dL]	0 (0%)	9.8 [8.9–11.7]	9.9 [8.9–12.4]	9.4 [8.8–10.7]	.159	.728
Platelets [G/L]	0 (0%)	152 [74–235]	157 [73–246]	121 [76- 177]	.302	.903
CRP [mg/L]	0 (0%)	118 [54–201]	118 [54–194]	131 [59–231]	.446	.231
PCT [ng/mL]	21 (11%)	0.97 [0.30–4.04]	0.71 [0.28–3.44]	1.89 [0.7–7.26]	.**023**	.519
Bilirubin [mg/dL]	0 (0%)	0.68 [0.40–1.64]	0.66 [0.39–1.45]	0.99 [0.50–3.23]	.106	.336
Creatinine [mg/dL]	0 (0%)	1.25 [0.80–7.00]	1.23 [0.80–7.00]	1.26 [0.78–7.00]	.639	.308
Mycological findings						
Serum GM (ODI)	0 (0%)	0.51 [0.14–0.90]	0.51 [0.14–0.87]	0.41 [0.14–0.95]	.890	.430
BAL GM (ODI)	35 (20%)	4.89 [1.88–7.45]	4.29 [1.75–7.14]	7.38 [2.91–8.1]	.**014**	.862
Β-D-Glucan pg/mL	5 (3%)	83.3 [15.4–255.7]	75.2 [15.4–251.2]	183.6 [75.4–320.2]	.**044**	.965
Asp-PCR	77 (44%)	44 (70%)	40 (70%)	4 (66%)	.859	.986
Asp-LFD	118 (66%)	39 (67%)	4 (66%)	35 (67%)	.975	.440
BAL culture	68 (38%)	/	/	/	.845	.275
*Aspergillus fumigatus*	/	100 (92%)	85 (92%)	15 (100%)	/	/
*Aspergillus flavus*	/	1 (1%)	1 (1%)	0 (0%)	/	/
*Aspergillus calidoustus*	/	1 (1%)	1 (1%)	0 (0%)	/	/
*Aspergillus niger*	/	4 (4%)	4 (4%)	0 (0%)	/	/
*Aspergillus terreus*	/	2 (2%)	2 (2%)	0 (0%)	/	/
Classification	0 (0%)	/	/	/	.286	.064
EORTC-MSG	/	64 (36%)	53 (35%)	11 (45%)	**/**	
FUNDICU	/	102 (58%)	89 (58%)	13 (55%)	/	/
No classification	/	11 (6%)	11 (7%)	0 (0%)	/	/
Fungal prophylaxis	100 (0%)	20 (11%)	19 (12%)	1(4%)	.235	.108
ICU characteristics						
ICU admission	0 (0%)	137 (77%)	114 (75%)	23 (95%)	.**020**	.229
APACHE II score	0 (0%)	21 [7–30]	19 [3–29]	30 [23–33]	.**002**	.109
SOFA	0 (0%)	7 [4–9]	7 [4–8]	10 [7–12]	**<**.**001**	.413
PaO_2_/FiO_2_	0 (0%)	126 [81–268]	127 [81–350]	105 [79–166]	.165	.361
Ventilatory support	0 (0%)	/	/	/	.464	.361
O_2_ supply only	0 (0%)	40	39	1	**/**	/
*HNFC*	0 (0%)	1	1	0	/	/
*NIV*	0 (0%)	13	12	1	/	/
*IV*	0 (0%)	101	81	20	/	/
*vv-ECMO*	0 (0%)	22	20	2	/	/

Data are reported as medians [25th–75th percentile] or as absolute counts (%). *P* denotes *P* values before ITPW weighting, *P*_IPTW_ denotes *P* values after IPTW adjustment. *P* values are either from rank-sum tests, χ^2^ tests, or Fisher's exact tests, as appropriate.

Abbreviations: APACHE II, acute physiology and chronic health evaluation II; Asp, aspergillus; BAL, broncho alveolar lavage; BMI, body mass index; GM, galactomannan; ICU, intensive care unit; LFD, lateral flow device; ODI, optical density index; PCR, polymerase chain reaction; SOFA, sequential organ failure assessment.

### Propensity Score

As previously outlined, patients in the echinocandin group had more severe disease (ie, higher SOFA and APACHE II scores), with more patients requiring ICU admission. They also showed differences in fungal biomarkers as well as inflammatory biomarkers toward more severity in the echinocandin group. These imbalances suggest a nonrandom assignment bias, possibly due to a preference for echinocandins in more critically ill patients to avoid possible triazole toxicity.

To address this imbalance, we pre-specified a propensity score model with a maximum of 10 variables. Since some laboratory values and severity scores were nested, we could reduce this to a 5-variable logistic regression model (Supplementary Table 1). The resulting propensity score, which covers the full probability range, was transformed into an IPTW. (Supplementary Figure 3) This approach effectively eliminated differences between the treatment groups after re-weighting, correcting for nonrandom assignment bias and yielding more reliable results. (Table 1; Supplementary Table 2 and Figure 4) The trimmed IPTW demonstrated similar success in reducing between-group differences, underscoring the robustness of the computed propensity score (data not shown).

### Selection of First-Line Antifungal Treatment and 30-Day OS

Within 30 days of IPA diagnosis, 73 deaths occurred, with ICU survival rates of 90% (95% CI 84–93) at 5 days, 70% (63–76) at 15 days, and 59% (51–76) at 30 days for the entire cohort (Supplementary Figure 5).

**Figure 1.**
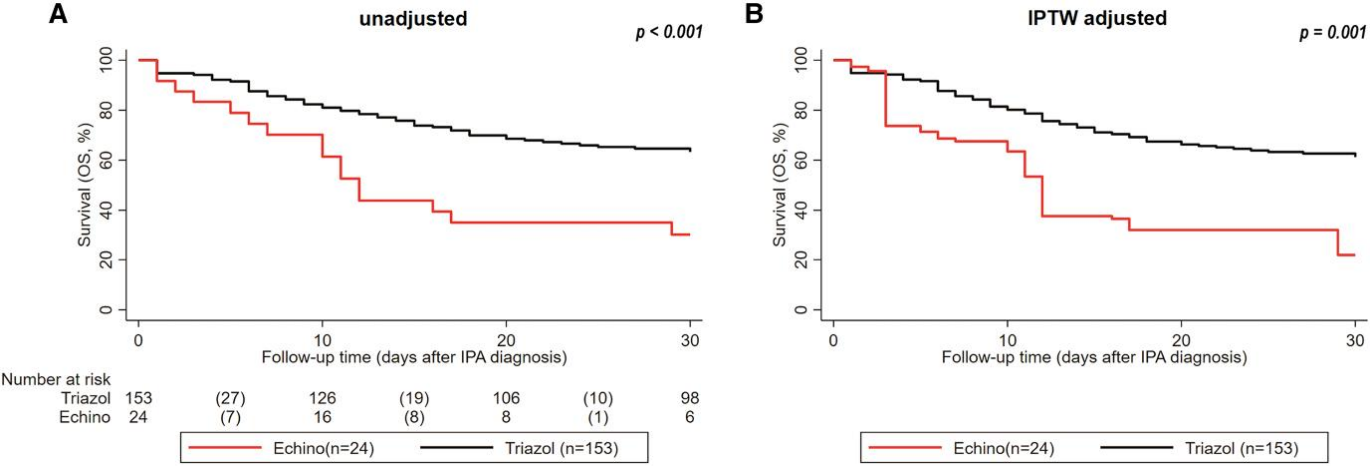
Thirty-day survival according to first-line antifungal treatment. *A*, Unadjusted analysis of the crude data. *B*, IPTW adjusted analysis. *P* values are calculated using the log-rank test. Risk table was only computed for the unadjusted analysis. Risk tables are shown only for the crude analysis, as IPTW adjustment yields uninterpretable patient counts.

In time-to-event analysis, patients receiving echinocandins as first-line treatment for IPA showed significantly worse 30-day OS: OS rates at 5, 15, and 30 days were 79% (95% CI 57–91), 44% (24–62), and 30% (13–50), respectively, compared to 91% (86–95), 74% (66–80), and 63% (55–70) in those treated with triazoles. Kaplan–Meier analysis confirmed significant differences between treatment groups (log-rank *P* < .001) (Figure 1*A*).

To address nonrandom assignment bias, we re-weighted the data using IPTW. Even after re-weighting, the worse survival outcomes for patients on first-line echinocandins persisted (log-rank *P* = .001) (Figure 1*B*).

We further assessed the impact of echinocandin first-line treatment on long-term survival of IPA treatment, extending the analysis to 90 days. The significant survival disparity remained in this analysis as well (log-rank *P*_crude_ = .001, log-rank *P*_IPTW_ = .003) (Supplementary Figure 6).

### Uni- and Multivariable Predictors of 30-Day OS

Univariable analysis of 30-day OS identified several factors associated with worse outcomes, including lower platelets, higher C-reactive protein (CRP), higher procalcitonin, elevated bilirubin and creatinine, high serum GM, negative *Aspergillus* culture from BAL, ICU admission, and higher APACHE II scores (Supplementary Table 3).

To evaluate the independent prognostic value of echinocandin as first-line IPA treatment for 30-day OS, multivariable analysis was performed, incorporating all significant univariable predictors. Procalcitonin (nested within CRP) and bilirubin, creatinine, and ICU admission (nested within APACHE II) were excluded from the final model. The negative prognostic impact of echinocandin first-line treatment use for IPA on 30-day survival remained significant with a hazard ratio (HR) of 1.88 (95% CI 1.05–3.37, *P* = .033) (Supplementary Table 4).

### Antifungal Treatment Trajectories

**Figure 2.**
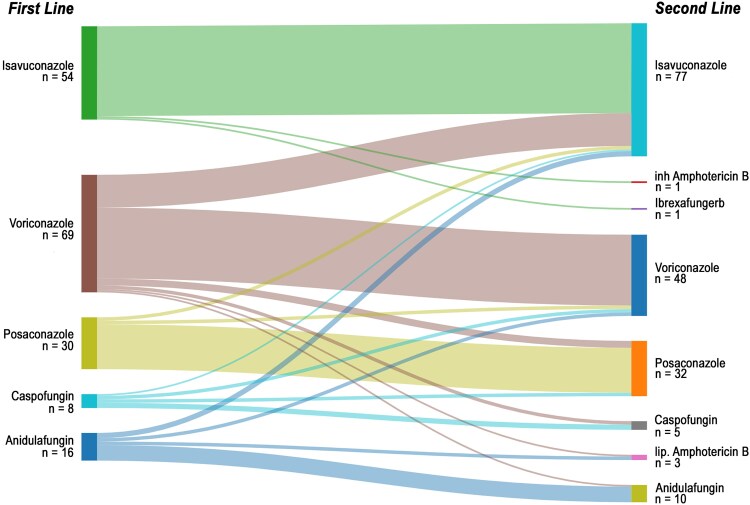
Sankey diagram depicting the treatment trajectories. On the left, first-line treatment agents are shown. Trajectories indicate patient switches to respective second-line therapies, with colors representing the first-line agent. The right side of the Sankey diagram shows the corresponding second-line substances.

Having shown that echinocandin as first-line antifungal treatment is associated with poor survival in our IPA cohort, we further analyzed treatment trajectories for specific antifungal agents. Most patients in the triazole group were treated with voriconazole (*n* = 69), followed by isavuconazole (*n* = 54) and posaconazole (*n* = 30). In the echinocandin group, caspofungin (*n* = 8) and anidulafungin (*n* = 16) were used (Figure 2).

Analysis of treatment changes revealed that switches were most frequent among patients receiving voriconazole, with 28 of 69 (41%) transitioning to a second-line agent. A total of 19/28 of these changes (68%) were due to side effects, identifying voriconazole as the most toxic mold-active agent in the cohort. The remaining 9 patients were switched from voriconazole due to treatment failure (3 cases) and insufficient trough levels (6 cases). Isavuconazole was switched due to treatment failure in one case and the identification of intrinsically azole-resistant *Aspergillus coledoustus* in another. Posaconazole was switched in 2 cases due to treatment failure, in 1 case due to insufficient trough levels, and 1 case due to drug–drug interactions. Echinocandins were generally well tolerated; however, treatment failure led to changes in 5 of 8 cases with caspofungin and 7 of 16 cases with anidulafungin. Other reasons for treatment changes are detailed in Table 2.

**Table 2.** Reasons for Treatment Change

	Toxicity	Treatment Failure	TDM Not Yielded	Other Reason	Change Total
Posaconazole (*n* = 30)	0 (0%)	2 (6%)	1 (3%)	1 (3%)	4 (13%)
Isavuconazole (*n* = 54)	0 (0%)	2 (4%)	0 (0%)	0 (0%)	2 (4%)
Voriconazole (*n* = 69)	19 (28%)	3 (4%)	6 (8%)	0 (0%)	28 (40%)
Caspofungin (*n* = 8)	0 (0%)	5 (63%)	0 (0%)	0 (0%)	5 (63%)
Anidulafungin (*n* = 16)	0 (0%)	7 (44%)	0 (0%)	0 (0%)	7 (44%)

### Echinocandin Treatment Switch Fails to Reverse Negative Survival Impact as First-Line Therapy

Finally, we explored whether switching from an echinocandin to another mold-active antifungal agent could reverse or mitigate its negative impact on survival. Among patients initially treated with echinocandins, 12 were switched to salvage therapy at a median of 8 days [IQR 6–9] due to treatment failure. Of these, 10 were switched to triazole treatment (5 isavuconazole 3 voriconazole, 2 posaconazole) and 2 to liposomal amphotericin B (Figure 2).

**Figure 3.**
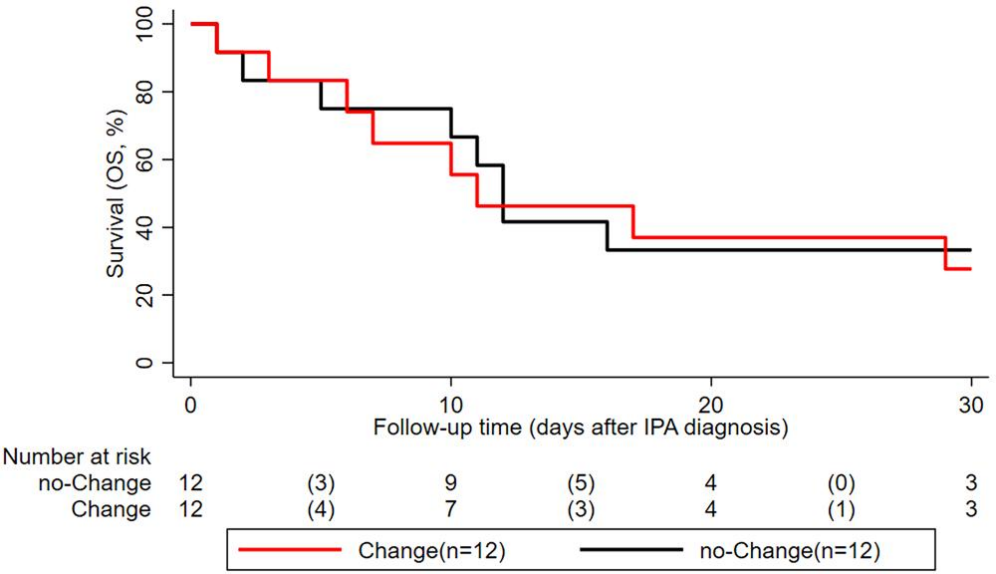
Thirty-day survival of patients sustaining on echinocandins versus treatment change. Change indicates a switch to either a triazoles or liposomal amphotericin B; no-change indicates sustained treatment with an echinocandin.

The 30-day OS rates for patients who were switch from an echinocandin to other mold-active antifungal agents were 83% [48–95] at 5 days, 46% [17–71] at 15 days, and 28% [6–54] at 30 days. Similarly, patients who remained on echinocandin treatment had OS rates of 75% [41–91], 42% [15–66], and 33% [10–59], respectively. Survival outcomes between the two groups were not significantly different (log-rank *P* = .86) (Figure 3).

The comparable survival rates persisted in the long-term 90-day OS analysis, which also showed no survival benefit for patients who were switched to alternative mold-active antifungal agents (log-rank *P* = .68) (Supplementary Figure 7).

## DISCUSSION

In this study, we used a propensity score–weighted analysis with the IPTW method to evaluate the efficacy of echinocandins as a first-line treatment for IPA in comparison to triazoles.

Echinocandins are an appealing option for IPA due to their good tolerability and safety profile. Although rarely used for treatment of IPA in clinical practice, they remain a recommended alternative in certain circumstances [4]. In our cohort, 24 patients (14%) received echinocandins as first-line therapy based on decisions of the treating physicians, while 153 (86%) were treated with triazoles. The rate of 14% is notably higher compared to 5% recently reported in a study on IPA treatment pathways [7]. The higher rate in our cohort might be explained by the high proportion of critically ill patients. Factors like including extracorporeal procedures, impaired liver function, or extensive multi-drug regimens with high interaction potential with triazoles obviously led physicians to choose echinocandins as the initial IPA treatment [7, 20–22]. We also observed a high proportion (19/69; 23%) of patients requiring a change in triazole treatment due to toxicity in our cohort, mainly among those treated with voriconazole.

In addition to the attractive echinocandine safety profile, the emergence of triazole resistance in some regions may lead physicians to consider echinocandin treatment [23]. However, in our cohort, only one patient required a switch from triazole first-line therapy due to resistance. This patient had IPA due to *Aspergillus calidoustus*, which is resistant to triazoles, and was subsequently treated with ibrexafungerp [24].

Although several preclinical studies and animal models support the use of echinocandins for IPA treatment, clinical trials in humans remain scarce [25, 26]. A trial comparing caspofungin and amphotericin B for empirical antifungal treatment in neutropenic fever found similar success rates using a composite endpoint. Caspofungin showed higher success rates among patients with baseline fungal infections, but the limited number of IA cases (12 per arm) precluded definitive conclusions [27]. Three phase 2 studies evaluated caspofungin as first-line therapy for proven or probable IA in patients with hematological malignancies, reporting success rates of 30%–55% [11, 12]. Other data on caspofungin efficacy primarily came from retrospective analyses or observational registries. A review estimated pooled success rates of 54% for first-line and 47% for second-line or salvage therapy, with rates varying widely (27%–92% for first-line, 28%–71% for salvage) [14, 27]. Notably, few studies directly compared caspofungin with other antifungals; only one reported significantly higher IA-associated mortality with caspofungin versus voriconazole [28]. Micafungin was also assessed in a few studies, showing similar success rates of 30%–50% [29, 30]. Therefore, published data suggests that the efficacy of echinocandins in IPA treatment is relatively consistent across different agents, making them suitable for group analysis.

In our study, patients receiving echinocandin as first-line treatment had significantly worse 30-day OS compared to those treated with triazoles. Specifically, the 30-day OS rates were 30% (95% CI 13–50) for the echinocandin group and 63% (55–70) for the triazole group. To address potential nonrandom assignment bias in our retrospective cohort, we applied propensity score matching. However, given the rarity of IPA and our small sample size, we utilized the IPTW method to avoid losing cases [31]. This finding persisted even after propensity score adjustment. Our analysis showed that echinocandins should be used with caution as first-line treatment of IPA, as they impaired outcomes for IPA patients. Furthermore, in a multivariable Cox model, echinocandin treatment was an independent negative prognostic factor, with a HR of 1.88 for worse 30-day OS, indicating a significant impairment in survival outcomes.

To prove the sustaining impact of echinocandin use on survival, we evaluated whether switching antifungal treatments from an echinocandin to a triazole or liposomal amphotericin B could reverse the negative impact of initial echinocandin use on the survival of IPA patients. Surprisingly, changing the echinocandin to another antifungal agent did not mitigate the adverse effects on survival. These findings further suggest avoiding echinocandins in the first-line treatment of IPA.

Recently, novel antifungal agents, including first-in-class options like olorofim, fosmanogepix, and ibrexafungerp, have emerged. Their availability probably diminishes the need for echinocandins as alternatives to triazoles in the treatment of IPA [6].

The present study cannot provide a comprehensive assessment of the use of novel echinocandins, such as rezafungin, in treating IPA. However, recent preclinical data suggest sufficient anti-mold activity. Combined with its favorable long-lasting and stable pharmacokinetics, rezafungin is considered a promising but yet underexplored potential mold treatment option [32].

To the best of our knowledge, this is the first study using a propensity score matching approach to address the potential role of echinocandins as first-line IPA treatment, analyzing 177 patients and contributing valuable evidence to the field. The results highlight the potentially limited role of echinocandins in the first-line treatment of IPA, suggesting a need for reevaluation of their place in routinely applied IPA treatment strategies and usage based on eg, potential triazole toxicity only.

While our study is retrospective and observational in nature, we employed propensity score matching to create comparable groups and enhance the reliability of our findings. However, such a design carries an inherent risk of bias that must be considered when interpreting the results. Despite including numerous variables in the propensity score model, some factors may not have been accounted for, potentially influencing the effect size. While our study employed IPTW to adjust for measured confounders, the higher early mortality observed in the echinocandin group compared to the azole group may reflect residual confounding by unmeasured or inadequately measured factors. This residual bias can persist even after IPTW adjustment, although the method assumes that all relevant confounders are included and correctly specified [33]. Our study is additionally limited by the relatively small number of patients in the echinocandin group, which reduces statistical power and may affect the precision of effect estimates [31].

Taken together, our data suggest that using echinocandins as first-line treatment for IPA may lead to poorer survival rates.

## Supplementary Material

ofaf709_Supplementary_Data

## Supplementary Data

Supplementary materials are available at *Open Forum Infectious Diseases* online. Consisting of data provided by the authors to benefit the reader, the posted materials are not copyedited and are the sole responsibility of the authors, so questions or comments should be addressed to the corresponding author.
