# Supplementary material for: Triazoles Versus Echinocandins for the First-Line Treatment of Invasive Pulmonary Aspergillosis: A Propensity Score–Weighted Multicenter Study
Source: Open Forum Infect Dis. 2025 Nov 18;12(12):ofaf709. doi: 10.1093/ofid/ofaf709 (PMC12696377; doi:10.1093/ofid/ofaf709)
Supplement: ofaf709_Supplementary_Data [file ofaf709_supplementary_data.docx]

Supplementary

**Triazoles versus Echinocandins for the first-line treatment of invasive pulmonary aspergillosis – A propensity score weighted multicenter study**

*Hatzl S. et al.*

**Supplementary Methods:**

**Propensity score:**

The propensity score (*e*) was derived from a multivariable logistic regression model, with early antifungal treatment, where all different triazols (eg. Posaconazole, voriconazole, isavuconazol) were combined to a single variable triazol and all echinocnadins (eg caspofungin, anidulafungin) as the outcome variable. To ensure at least three events per predictor variable, we limited the model to a maximum of 10 predictors. Variables were selected if they had a *p*-value ≤ 0.15 and/or an SMD ≥ 0.30. The final multivariable logistic regression model consisted of 5 variables listed in **Supplementary Table 1**.

The propensity score (*e*) was then transformed into an inverse-probability-of-treatment-weight (*IPTW*) using the average treatment effect principle:

*IPTW* $=\frac{( antifungal treatment)}{e}+\frac{1-(antifungal treatment)}{1-e}$ where administration of a triazole represents the treatment assignment. [1, 2]

| **Center** | **Triazole** | **Echinocandin** |
| --- | --- | --- |
| LKH-Graz (Internal Medicine) | 77 | 13 |
| LKH-Graz (Surgery) | 16 | 2 |
| LKH-Graz (Neurology) | 5 | 0 |
| LKH-Oststeiermark (Mixed) | 14 | 3 |
| LKH-Südsteiermark (Mixed) | 14 | 1 |
| LKH-Weststeiermark (Mixed) | 12 | 2 |
| LKH-Hochsteiermark (Mixed) | 3 | 0 |
| LKH-Graz II (Mixed) | 12 | 2 |
| LKH-Rottenmann/ Bad Aussee (Mixed) | 0 | 1 |


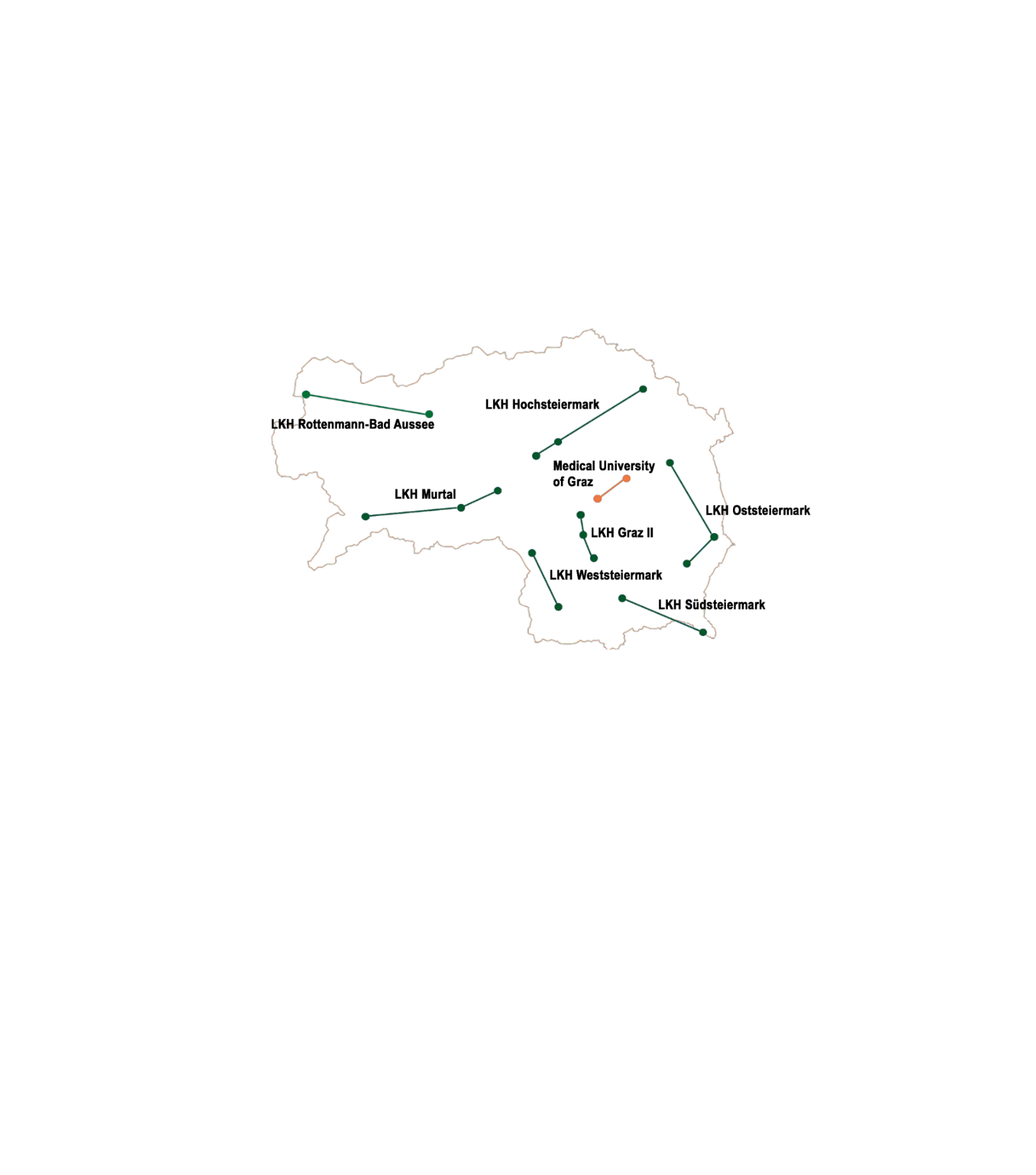


**Supplementary Figure 1: Treatment centers involved in the study**

Each point in the figure refers to a single hospital. The connecting lines show the hospital-networks. LKH – hospital network, n= number of participants enrolled by each center. The number in each category indicates the number of patients included in each treatment center.


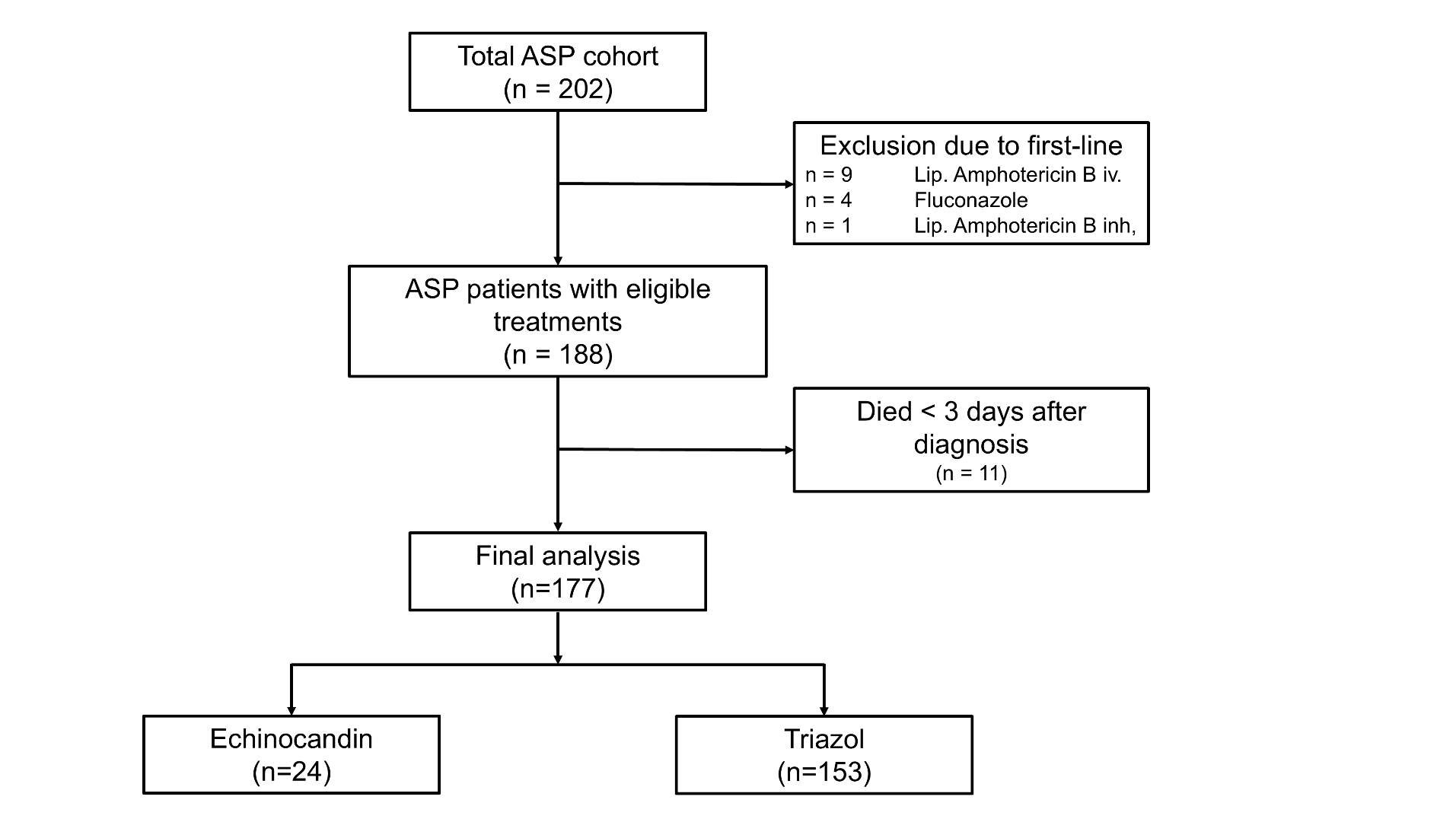


**Supplementary Figure 2: Flow chart**

The figure depicts the inclusion of patients in the final analysis (N=177) from the total cohort of 202 patients with IPA


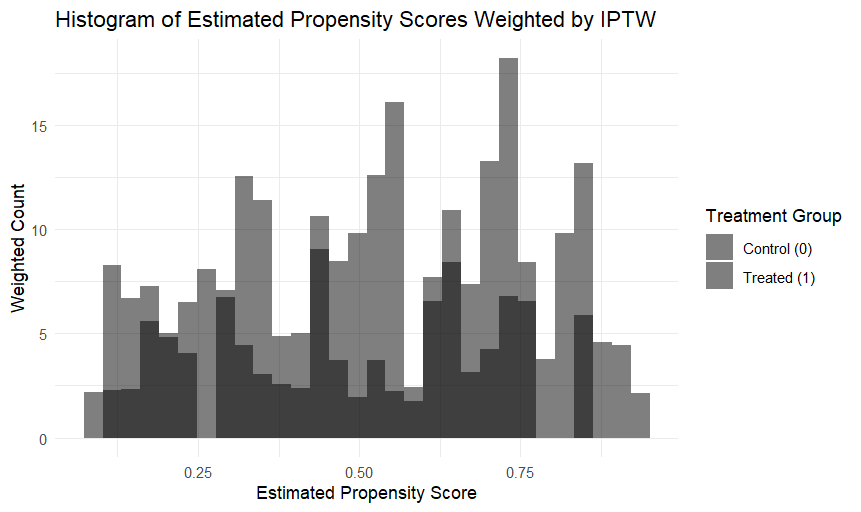

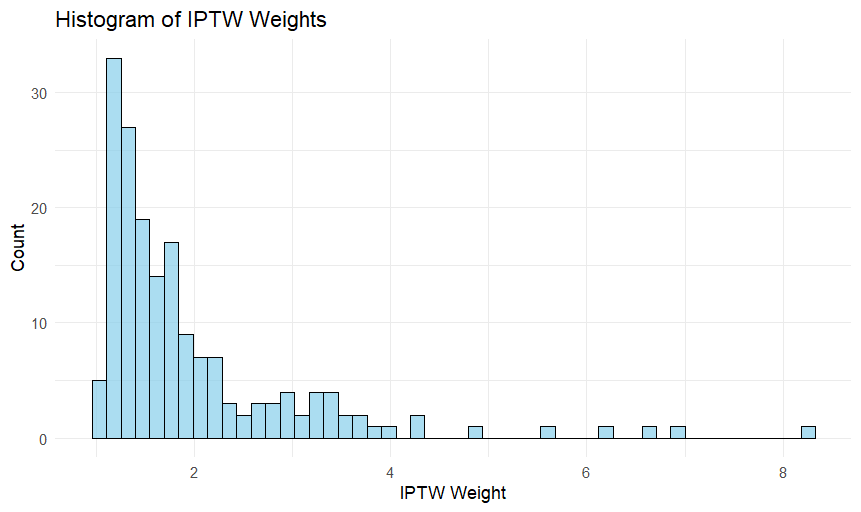
 **A** **B**

**Supplementary Figure 3: Histograms of the Propensity Score and the IPTW**.

(A) The propensity score can range from 0 to 1. Multiply by 100 to obtain probabilities (in percent) of having received echinocandin treatment. (B) The IPTW was defined as the inverse of the probability of receiving the treatment that the patient received


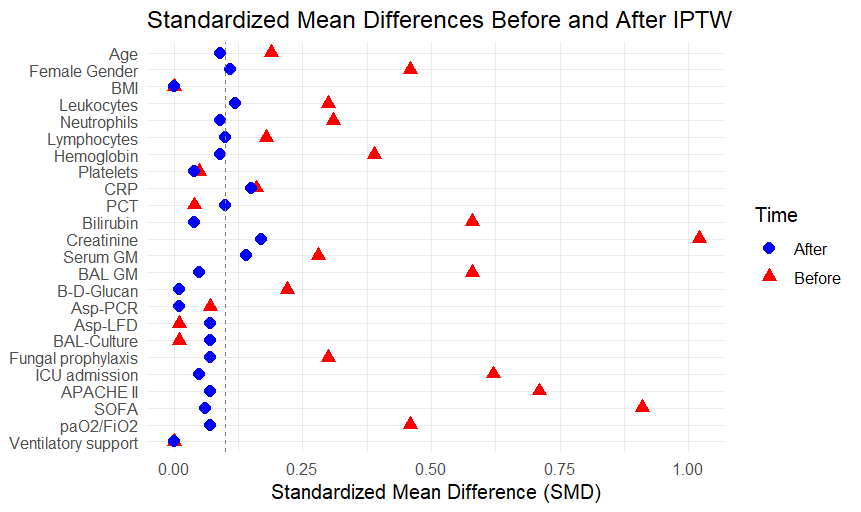


**Supplementary Figure 4: Standard mean difference Plot**

Red triangels show SMD´s before adjustment, blue dots show the SMD after IPTW adjustment. BMI – body mass index; GM – galactomannan; BAL – broncho alveolar lavage; PCR – polymerase chain reaction; Asp – aspergillus; LFD – lateral flow device; ODI – optical density index; ICU – intensive care unit; APACHE II – acute physiology and chronic health evaluation II; SOFA – sequential organ failure assessment, SMD – standard mean difference, IPTW – inverse probability of treatment weight


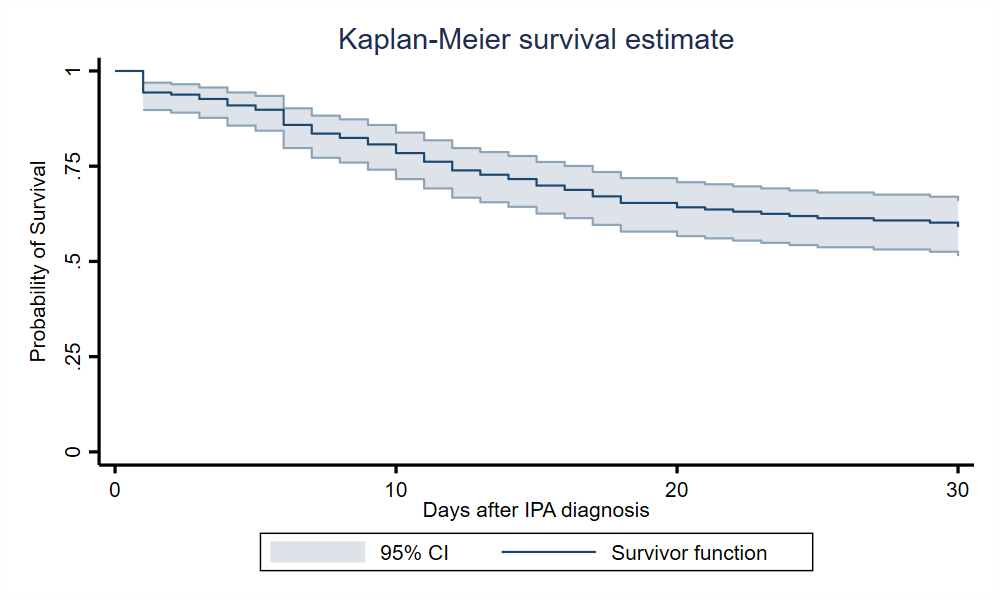


**Supplementary Figure 5: Survival of the whole cohort**

The figure shows the Kaplan-Meier curve displaying the survival function of the whole cohort

95% CI – 95% confidence interval


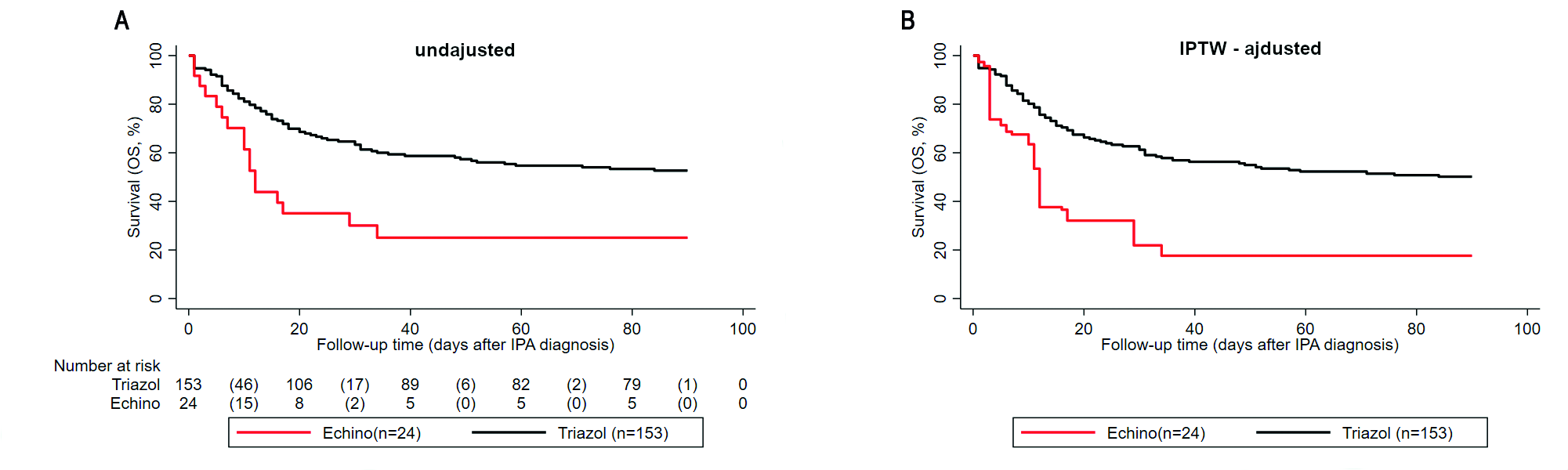


**Supplementary Figure 6: 90-day ICU survival according to first-line treatment (Long-term survival)**

A) Unadjusted analysis B) IPTW adjusted analysis.


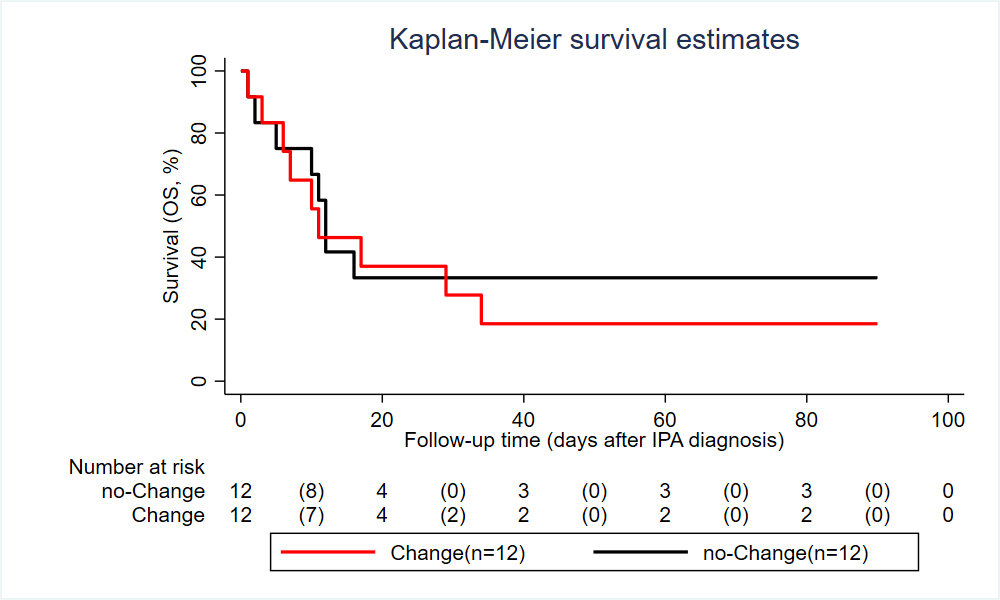


**Supplementary Figure 7: 90-day ICU survival according to second-line treatment switch (Long-term survival)**

Change indicates a switch to either a triazol or liposomal amphotericin B; no-Change indicates sustained treatment with an echinocandin

| **Variable** |  | **Multivariable Odds Ratio (OR)** | **95%CI** | **P** |
| --- | --- | --- | --- | --- |
|  |  |  |  |  |
| **Demographic variables** |  |  |  |  |
| Female Gender |  | 3.81 | 0.93-15.71 | 0.064 |
| PCT (per 1 unit increase) |  | 0.97 | 0.94-1.01 | 0.151 |
| BAL-GM (per 1 unit increase) |  | 1.15 | 1.00-1.33 | 0.040 |
| *β-D*-Glucan |  | 1.00 | 0.99-1.00 | 0.437 |
| SOFA-score (per 1 point increase) |  | 1.17 | 1.01-1.37 | 0.034 |

**Supplementary Table 1: A propensity score model for treatment group assignment**

We predicted a 5-variable propensity score model.

PCT- procalcitonin; BAL – broncho-alveolar lavage; GM – galactomannan; SOFA -sequential organ failure assessment.

| **Variable** | **Missing n (%)** | **SMD** | **SMD_IPTW_** |
| --- | --- | --- | --- |
| Age (years) | 0 (0%) | 0.19 | **0.37** |
| Female Gender | 0 (0%) | **0.46** | 0.24 |
| BMI (kg/m²) | 0 (0%) | 0.00 | 0.00 |
|  |  |  |  |
| **Laboratory findings** |  |  |  |
| Leukocytes [G/L] | 0 (0%) | 0.30 | 0.24 |
| Neutrophils [G/L] | 0 (0%) | **0.31** | 0.23 |
| Lymphocytes [G/L] | 0 (0%) | 0.18 | 0.15 |
| Hemoglobin [g/dL] | 0 (0%) | **0.39** | 0.09 |
| Platelets [G/L] | 0 (0%) | 0.05 | 0.04 |
| CRP [mg/L] | 0 (0%) | 0.16 | 0.15 |
| PCT [ng/ml] | 21 (11%) | 0.04 | 0.10 |
| Bilirubin [mg/dL] | 0 (0%) | **0.58** | 0.25 |
| Creatinine [mg/dL] | 0 (0%) | **1.02** | 0.31 |
|  |  |  |  |
| **Mycological findings** |  |  |  |
| Serum GM (ODI) | 0 (0%) | 0.28 | 0.29 |
| BAL GM (ODI) | 35 (20 %) | **0.58** | 0.05 |
| Β-D-Glucan pg/mL | 5 (3%) | 0.22 | 0.01 |
| Asp-PCR | 77 (44%) | 0.07 | 0.01 |
| Asp-LFD | 118 (66%) | 0.01 | 0.07 |
| BAL-Culture | 68 (38%) | 0.01 | 0.21 |
|  |  |  |  |
| **Fungal prophylaxis** | 100 (0%) | 0.30 | 0.16 |
|  |  |  |  |
| **ICU-Characteristics** |  |  |  |
| ICU admission | 0 (0%) | **0.62** | 0.14 |
| APACHE II score | 0 (0%) | **0.71** | 0.24 |
| SOFA | 0 (0%) | **0.91** | 0.18 |
| paO_2_/FiO_2_ | 0 (0%) | **0.46** | 0.26 |
| Ventilatory support | 0 (0%) | 0.00 | 0.00 |

**Supplementary Table 2: Standard mean difference (SMD)**

SMD_IPTW_ indicates the SMD value after IPTW adjustment. Values > 0.3 were marked bold.

BMI – body mass index; GM – galactomannan; BAL – broncho alveolar lavage; PCR – polymerase chain reaction; Asp – aspergillus; LFD – lateral flow device; ODI – optical density index; ICU – intensive care unit; APACHE II – acute physiology and chronic health evaluation II; SOFA – sequential organ failure assessment.

| **Variable** | **n** | **HR [95%CI]** | **p-value** |
| --- | --- | --- | --- |
| Echinocandin as first-line treatment | 177 | 2.58 [1.47-4.51] | **>0.001** |
|  |  |  |  |
| Age per 10 years | 177 | 1.02 [0.85-1.23] | 0.82 |
| Female | 177 | 0.90 [0.55-1.48] | 0.68 |
| BMI per 5 kg/m² | 177 | 1.06 [0.86-1.32] | 0.55 |
|  |  |  |  |
| **Laboratory findings** |  |  |  |
| Leukocytes per 5 G/L increase | 177 | 1.09 [0.95-1.25] | 0.20 |
| Neutrophils per 5 G/L increase | 177 | 1.12 [0.97-1.29] | 0.10 |
| Lymphocytes per 5 G/L increase | 177 | 0.99 [0.60-1.65] | 0.98 |
| Haemoglobin per 5g/dL increase | 177 | 0.67 [0.38-1.18] | 0.17 |
| Platelets per 50 G/L decrease | 177 | 1.17 [1.05-1.31] | **0.005** |
| CRP per 100 mg/L increase | 177 | 1.19 [1.00-1.41] | **0.039** |
| PCT per 10 ng/ml increase | 177 | 1.09 [1.00-1.19] | **0.047** |
| Bilirubin per mg/dL increase | 177 | 1.05 [1.01-1.10] | **0.011** |
| Creatinine per mg/dL increase | 177 | 1.14 [1.05-1.24] | **0.001** |
|  |  |  |  |
| **Mycological findings** |  |  |  |
| Serum GM (ODI) | 177 | 1.23 [1.09-1.39] | **0.001** |
| BAL GM (ODI) | 142 | 1.06 [0.99-1.12] | 0.06 |
| Β-D-Glucan per 500 pg/mL | 177 | 1.19 [1.02-1.39] | 0.05 |
| Asp-PCR | 63 | 1.04 [0.48-2.26] | 0.90 |
| Asp-LFD | 58 | 1.22 [0.53-2.79] | 0.63 |
| BAL-Culture | 174 | 0.58 [0.37-0.93] | **0.024** |
|  |  |  |  |
| **Immunosuppression** |  |  |  |
| Neutropenia | 177 | 1.28 [0.67-2.44] | 0.45 |
| Haematological Malignancy | 177 | 1.05 [0.58-1.92] | 0.85 |
| Corticoids | 177 | 1.10 [0.65-1.89] | 0.70 |
|  |  |  |  |
| **ICU-characteristics** |  |  |  |
| ICU admission | 177 | 6.55 [2.39-17.99] | **>0.001** |
| APACHE | 177 | 1.23 [1.13-1.35] | **>0.001** |

**Supplementary Table 3: Univariable predictors of 30-day survival**

BMI – body mass index; GM – galactomannan; BAL – broncho alveolar lavage; PCR – polymerase chain reaction; Asp – aspergillus; LFD – lateral flow device; ODI – optical density index; ICU – intensive care unit; APACHE II – acute physiology and chronic health evaluation II;

| **Variable** | **HR [95%CI]** | **p-value** |
| --- | --- | --- |
| Echinocandin as first-line treatment | 1.88 [1.05-3.37] | **0.033** |
| C-reactive protein | 1.00 [0.99-1.00] | 0.479 |
| Serum Galactomannan | 1.19 [1.04-1.35] | **0.007** |
| Aspergillus Culture from BAL | 0.60 [0.37-0.97] | **0.037** |
| APACHE II | 1.03 [1.01-1.05] | **>0.001** |

**Supplementary Table 3: Multivariable model of 30-day survival**

BAL – broncho alveolar lavage; APACHE II – acute physiology and chronic health evaluation II;

**Alt text section:**

Supplementary Figure 1:

Flow chart showing patient inclusion process for the final analysis, starting with 202 patients with invasive pulmonary aspergillosis and, after applying exclusion criteria, ending with 177 patients included in the final study cohort.

Supplementary Figure 2:

Two histograms illustrating (A) the distribution of propensity scores ranging from 0 to 1, representing the probability of receiving echinocandin treatment, and (B) the distribution of inverse probability of treatment weights (IPTW), calculated as the inverse of the probability of receiving the actual treatment given.

Supplementary Figure 3:

Standard mean difference plot comparing baseline covariate balance before and after IPTW adjustment. Red triangles indicate SMDs before adjustment; blue dots indicate SMDs after adjustment. Includes variables such as BMI, GM, BAL, PCR, Asp, LFD, ODI, ICU, APACHE II, and SOFA.

Supplementary Figure 4:

Kaplan–Meier curve showing the survival function of the entire patient cohort, with shaded areas representing the 95% confidence interval around the survival estimate over time.

Supplementary Figure 5:

Kaplan–Meier curves depicting 90-day ICU survival by first-line treatment type. Panel A shows unadjusted survival estimates; Panel B shows IPTW-adjusted estimates. Curves compare survival probabilities over time for each treatment group.

Supplementary Figure 6:

Kaplan–Meier curves showing 90-day ICU survival by first-line treatment. Panel A presents the unadjusted analysis; Panel B presents the IPTW-adjusted analysis. Survival probability is plotted over time for each treatment group.

**References:**

1. Chesnaye NC, Stel VS, Tripepi G, et al. An introduction to inverse probability of treatment weighting in observational research. Clin Kidney J, **2021**; 15: 14–20.

2. Austin PC, Stuart EA. Moving towards best practice when using inverse probability of treatment weighting (IPTW) using the propensity score to estimate causal treatment effects in observational studies. Stat Med, **2015**; 34: 3661–79.
